# Layer-Wise Relevance Analysis for Motif Recognition in the Activation Pathway of the *β*2-*Adrenergic* GPCR Receptor

**DOI:** 10.3390/ijms24021155

**Published:** 2023-01-06

**Authors:** Mario A. Gutiérrez-Mondragón, Caroline König, Alfredo Vellido

**Affiliations:** 1 Computer Science Department, Universitat Politècnica de Catalunya—UPC BarcelonaTech, 08034 Barcelona, Spain; 2 Intelligent Data Science and Artificial Intelligence (IDEAI-UPC) Research Center, Universitat Politècnica de Catalunya—UPC BarcelonaTech, 08034 Barcelona, Spain

**Keywords:** GPCRs, *β*2-adrenergic receptors, proteomics, molecular dynamics, signal pathways, deep learning, convolution networks, interpretability, layer-wise relevance

## Abstract

G-protein-coupled receptors (GPCRs) are cell membrane proteins of relevance as therapeutic targets, and are associated to the development of treatments for illnesses such as diabetes, Alzheimer’s, or even cancer. Therefore, comprehending the underlying mechanisms of the receptor functional properties is of particular interest in pharmacoproteomics and in disease therapy at large. Their interaction with ligands elicits multiple molecular rearrangements all along their structure, inducing activation pathways that distinctly influence the cell response. In this work, we studied GPCR signaling pathways from molecular dynamics simulations as they provide rich information about the dynamic nature of the receptors. We focused on studying the molecular properties of the receptors using deep-learning-based methods. In particular, we designed and trained a one-dimensional convolution neural network and illustrated its use in a classification of conformational states: active, intermediate, or inactive, of the *β2-adrenergic* receptor when bound to the full agonist BI-167107. Through a novel explainability-oriented investigation of the prediction results, we were able to identify and assess the contribution of individual *motifs* (residues) influencing a particular activation pathway. Consequently, we contribute a methodology that assists in the elucidation of the underlying mechanisms of receptor activation–deactivation.

## 1. Introduction

G-protein-coupled receptors (GPCRs) are a functionally relevant family of cell membrane proteins characterized by seven transmembrane alpha-helical structural regions connected by extra- and intra-cellular loops [1]. At the most basic level, the function of these receptors depends on their ability to change their form [2]. Therefore, understanding the dynamic nature of these complex structures has critical implications for both basic science and pharmacology [3]. In the latter context, most of the current druggable targets are proteins, and GPCRs in particular have been shown to play an important role in the development of treatments for several diseases [4]. For this reason, the study of the structural and physicochemical dynamics of these proteins and their role in their functional properties is of particular practical interest from a disease therapy research viewpoint.

The current understanding of the functional properties of many protein structures would be away from our comprehension without X-ray crystallography and spectroscopy methods [5]. These studies have been of paramount importance for capturing relevant information about the three-dimensional position of the atoms, providing a wealth of information for elucidating protein structures [6]. Nonetheless, the description of these receptors as rigid entities does not allow for a full appreciation of their dynamic nature. In this context, molecular dynamics (MD) simulations have been introduced as crucial analytical tools [7] for investigating the receptor intrinsic flexibility and conformational plasticity at the atomic level in very small timescales [8]. They are suitable for studying and complementing the functional mechanics of diverse molecular processes [9].

These computational techniques have evolved into essential tools for enriching molecular structural information [8]. Moreover, they are crucial for extending the understanding of several processes related to the receptor function, e.g., protein conformational diversity, binding pocket analysis, protein folding, ligand binding and its influence on the signaling process, among others (see [9,10,11,12,13]). Nonetheless, the investigation of the large amounts of protein information generated by MD is a far from a trivial challenge [14]. In this context, machine learning (ML) algorithms can provide a differential advantage for the analysis of the metadata produced. ML algorithms have been successful as analytical tools for healthcare and medicine in general and for bioinformatics in particular [15]. In the latter context, for instance, ML has been used for the analysis of the dynamics of protein pockets and the investigation of binding affinity. The application of ML to the prediction of binding sites is investigated in [16,17,18], to name a few. Other contributions have addressed the improvement of MD simulations: see, for instance, [19,20].

Despite their arguable success, ML algorithms involve the use of domain expertise for the correct data definition, to reduce complexity, and to optimize accuracy and precision. Manual feature engineering and human intervention may increase the risk of bypassing subtle structural transformations that could be relevant from the functional perspective. Alternatively, deep learning (DL)-based algorithms have emerged as critical tools for automatically learning complex patterns when the domain is particularly difficult. The use of these models has exponentially grown over the last decade in domains such as bioinformatics and medicine [21,22]), as well as in proteomics in particular. DL applications and their limitations in proteomics are discussed in [23,24,25], to cite a few. More specifically, some studies have used these methods in binding prediction problems [26,27]. Their use for the analysis and prediction of signaling pathways can be found, for instance, in [28,29,30].

Broadly, the research reviewed in the previous paragraphs reveals the potential of DL methods to extract valuable knowledge concerning the underlying mechanisms of the receptors. Nonetheless, the inherent lack of interpretability of DL approaches stands in the way of validation and the widespread use of such methods in this domain. These models exacerbate the *black box* problem often associated with shallow artificial neural networks, making it hard to explain their decisions. As a result of this shortcoming, the concept of explainable artificial intelligence (XAI) has become a line of research on its own for furthering trust in the prediction results, as mentioned in [31,32,33]. Though the *explainability* concept is highly domain-dependent, efforts have been made to formulate policies for establishing what explanations should be or entail [34]. In this sense, a taxonomy considering the scope, methodology, and usage for distinguishing explainability techniques is described in [35].

On the other hand, as mentioned, the investigation of DL interpretability in the study of proteomics research has gained interest in recent years. For instance, the study of conformational state changes induced by ligands using a sensitivity analysis was presented in [36]. The LIME-Local Interpretable Model-Agnostic Explanations algorithm was used in the investigation of relevant residues for denoting active and inactive states of GPCR receptors [37]. In this paper, we propose the use of the layer-wise relevance propagation (LRP) algorithm for exploring the conformational states (active, intermediate, and inactive) of a GPCR receptor with a supervised classifier in order to gain insights from the internal structure of the proposed DL-based model for producing robust and intuitive evidence regarding relevant aspects for the prediction of the different conformational states [38,39]. The LRP algorithm overcomes a limitation of the LIME algorithm as it is not directly based on a surrogate model [40]. The present study is novel as it uses, in the first place, nearly untransformed MD simulation data, which enable the exploration of a large amount of different intermediate states from the 3D position of their residues. Furthermore, the relevance analysis is extended to a multi-class classification problem as the interest is in the recognition of the active, intermediate, and inactive conformational state. In proteomics research, the LRP algorithm was valuable in [41,42,43] for studying protein–ligand interactions. Regarding the application of the LRP algorithm in other domains, it was successfully applied in the medical context for promoting trust and decision support for the proposed models in [44,45]. An overview and comparison between different XAI techniques can be found in [46,47,48].

In this study, we focus on investigating the underlying mechanisms of molecular activation–deactivation [10]. MD simulations are key to studying the dynamics of individual atoms over time. From this information, it is possible to reveal a virtually infinite number of conformations, both spontaneous and induced by a ligand-binding process [1]. These conformational states (clusters of conformations) cause intermediate re-orderings along different signaling pathways that influence a route to activate and deactivate the receptor and, thereby, condition their functional response [49].

The development of analytical tools that enable the study of the vast amount of generated data is a relevant research goal on its own. Here, we propose one such tool: a one-dimensional convolution neural network (1D-CNN), whose workings we illustrate by exploring the explicit representation of the β2-adrenergic ($\beta_{2}AR$) receptor provided by MD simulations. Importantly, we focus on its interpretability by investigating motifs (residues or groups of residues) associated with the conformation of signaling pathways that are relevant to distinguishing between conformational states. To this end, as previously mentioned, we put forward an explainable passive algorithm, based on relevant local patterns attribution (related to critical residues), for discriminating the receptor conformational states (active, intermediate, and inactive). Our approach also analyzes the specific contribution of each of the transmembrane regions (referred to as helix H1–H7), as well as those of its intracellular (ICL1–ICL3) and extracellular loops (ECL1–ECL3), to such a conformational state discrimination problem.

## 2. Results and Discussion

The classification problem in this study, as described in Section 3, entails separating three states: active, intermediate, and inactive. The results are summarized in Table 1. Overall, the model performance results in a 77.63% accuracy on the validation set.

**Figure 1:**
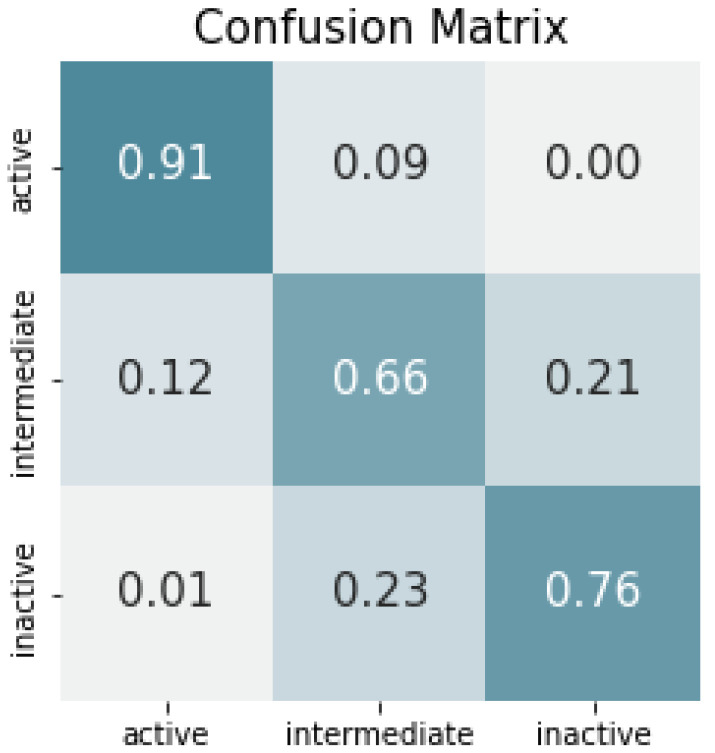
Normalized confusion matrix in the validation set.

In more detail, the confusion matrix displayed in Figure 1 reveals that the classifier is particularly good at discriminating the active states from both the inactive and the intermediate ones. Nevertheless, it is evident that it struggles to predict intermediate states as a separate class (with an F1 score of 66.66%). These results are consistent with other studies on the prediction of conformational states in GPCRs, where the intermediate state was the most difficult to predict, achieving less accurate results compared to the active and inactive states [50]. Most of these misclassifications concern intermediate states being predicted as inactive and *vice versa*. From this result, we could infer that the transition activation pathway from inactive to intermediate occurs very gradually, with barely perceptible re-orderings in the trajectory, i.e., the residue movement might be very limited compared to those involved in the transition from intermediate to active states.

This result could be somewhat expected, but it does not inform us about which parts of the molecule (that is, which motifs) are more relevant to the discrimination between states.

The interpretability study in a subsequent subsection will clarify the classifier decision-making process, providing valuable insights to ascertain the activation pathway of the structure.

### 2.1. Model Interpretability Using Relevance Values

As previously explained, we strived to achieve model interpretability in the reported experiments by implementing the LRP algorithm. Generally speaking, it operates in an artificial neural network model by scoring the contribution of its individual neurons by *backpropagating* the activation through the neural network until it reaches the input. An intuitive color map of the input space could then be produced, highlighting meaningful patterns for each conformational state.

The received contribution by a neuron is called *relevance* (*R*) and is redistributed equally in subsequent layers; the more a neuron contributes to the activation, the most relevance it receives. Thus, assuming *j* and *k* as the indices for two neurons in any consecutive layers, the *R* map can be computed following this basic rule:

(1) $$R_{j}=\sum_{k}\frac{a_{j}w_{jk}}{\sum_{0,j}a_{j}w_{jk}}R_{k}$$

From this equation, an initial relevance vector *R* is defined at the output layer, where each entry corresponds to the activation of one of the *C* classes (conformational states). Essentially, the formulation implies that the relevance computation of a neuron *j* is a consequence of its influence over all of the *k* neurons in the next layer. In particular, the numerator models the contribution of the neuron *j* to the neuron *k*, in which, $a_{j}$ denotes the neuron activation and $w_{jk}$ is the weight of the connection between two neurons. Likewise, to ensure the redistribution of the relevance, we must divide by the sum of the neuron contributions of the lower layer. Following this formulation, we iteratively scored each neuron in the neural network in order to produce explanations for the model predictions.

**Figure 2:**
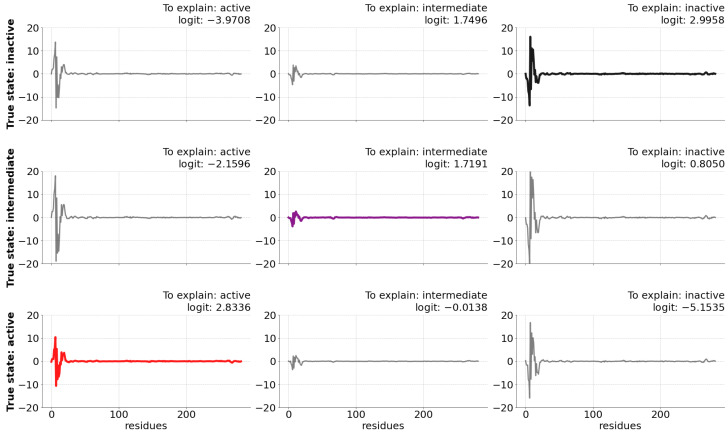
Example of use of LRP using just three frames, in which each row illustrates the relevance contribution (vertical axis) of each residue (horizontal axis) for the *active, intermediate, and inactive* states compared with the true state (as described in the left hand-side legends) of a given frame. The *logit* value denotes the raw predictions computed by the last layer of the neural network that are input to generate the model interpretation. Note that the *logit* value is highest for the explanation of the correct class prediction (the diagonal of the graph).

In this context, LRP enables the investigation of the reasons behind the model *class* predictions. As an illustration, Figure 2 displays explanations for each conformational state prediction in contrast with the true protein state using just three frames of a correctly predicted trajectory.

#### 2.1.1. Construction of Relevance Maps

We can achieve more intuitive relevance maps by using the so-called *propagation rules* described in [51], which are designed to penalize or emphasize the contribution of the neurons. Importantly, the decision to adopt any rule must consider the motivations behind the need of model explanations. In the context of our analysis, providing confidence about the system learning the correct features to identify a particular object in the input is only of relative importance. Instead, we are seeking all of the patterns in the MD trajectory that could be relevant to the protein function. For this reason, the epsilon rule (LRP-ϵ) was preferred in this study to prevent overly complex explanations. The LRP-ϵ rule adds a small and positive constant to the denominator in Equation (1) to take the form:

(2) $$R_{j}=\sum_{k}\frac{a_{j}w_{jk}}{\epsilon +\sum_{0,j}a_{j}w_{jk}}R_{k}$$

avoiding weak and noisy mappings, thus inducing a trustworthy interpretation. Other rules were designed to stress positive contribution, therefore generating a more detailed explanation (for instance, in the image analysis domain).

**Figure 3:**
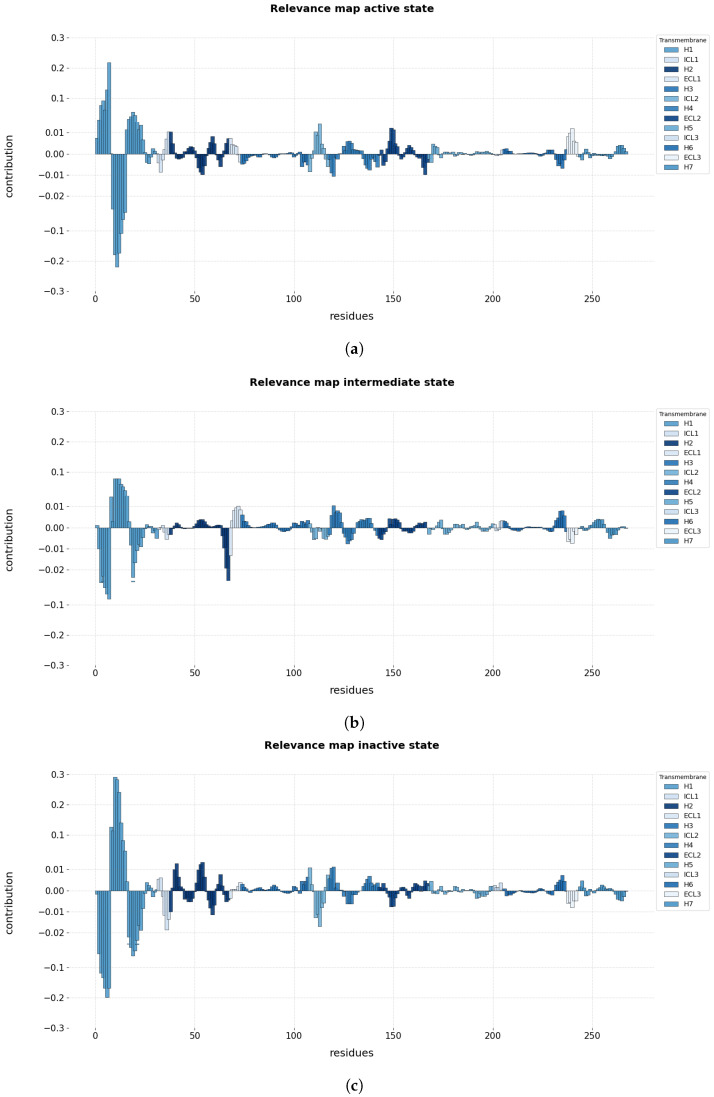
Computed average relevance of the residues to conformational states in 100 randomly selected trajectories selecting individual frames correctly predicted. The added relevance is color-coded according to the transmembrane helices (H1–H7) and the intracellular (ICL1–ICL3) and extracellular (ECL1–ECL3) loops. (**a**) Relevance map illustrating the contribution of residues for the active state, (**b**) relevance map illustrating the contribution of residues for the intermediate state, and (**c**) relevance map illustrating the contribution of residues for the inactive state.

According to this, the average relevance contribution of the residues for the prediction of a conformational state using the LRP-ϵ rule is displayed in Figure 3. It must be emphasized that we made inferences over 100 randomly chosen trajectories, selecting individual frames (in equal amount per conformational state) correctly predicted to compute explanations of the predictions results.

Importantly, to ease the analysis while being consistent with the original dimension of the data, the *R* maps were calculated for each residue in a particular trajectory by summing the individual relevance per coordinate (XYZ) in the center of mass, and then dividing by the number of coordinates. Then, the final *R* maps explaining each conformational state were calculated by averaging the computed relevance from each residue in the 100 chosen trajectories.

From the relevance maps reported in Figure 3, it is possible to gain a coarse understanding about the overall contribution of the different regions of the receptor for the prediction of each conformational state. Figure 3a describes the relevance values for the prediction of the active state. The relevance map highlights H1 as having the highest positive and negative values for the prediction of the active state, achieving maximum absolute values in the region of 0.2. The remaining regions also show positive and negative contributions to the prediction of the target class, but their relevance values are notably lower than that of H1. Figure 3b shows the relevance map for the prediction of the intermediate state. The map again highlights H1 with both positive and negative values, this time with a somehow lower magnitude in the area of 0.05 at most, and H3 with locally high negative relevance values. The remaining regions show both negative and positive contributions, but reaching comparable low values. Finally, Figure 3c shows the relevance map for the prediction of the inactive state. The map shows that the H1 region has very high positive and negative contributions, reaching absolute values close to 0.30. Interestingly, the ICL1 and ICL2 regions also show quite high negative relevance values. The remaining regions contribute with lower relevance values both to the positive or negative prediction.

#### 2.1.2. Local Relevance: Key Residues and Motifs

**Figure 4:**
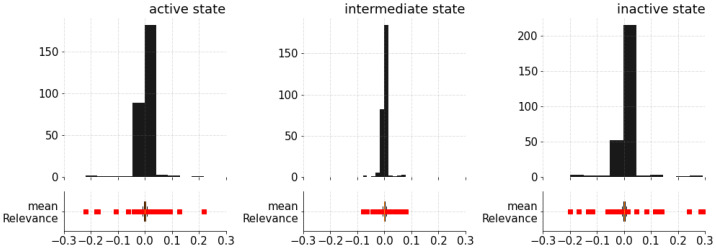
Histograms of the computed relevance contributions for each of the conformational states.

Figure 3 makes clear that some specific residues in Helix 1 (H1) contribute the most (are the most relevant) to predicting the conformational states. Lesser but still clear contributions reflecting transformations in the protein structure that influence the final prediction can be seen in the remaining regions. From this visualization, it is difficult to recognize motifs in the trajectory that contribute positively (i.e., with positive relevance) to an active state but negatively to an inactive state. To assess relevant contributions (motifs) for predicting a conformational state, a simple statistical analysis on the distribution of the calculated relevance for each conformational state was carried out (Figure 4).

**Figure 5:**
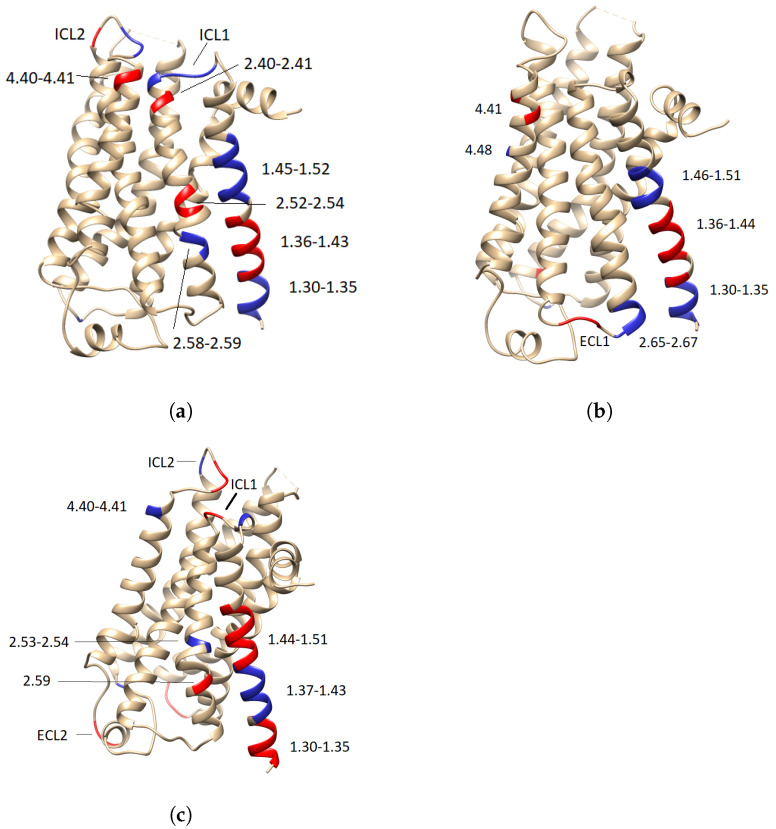
Conformational states of the receptor highlighting the residues with positive and negative contribution in red and blue, respectively. (**a**) Active state, (**b**) intermediate state, and (**c**) inactive state.

From the histograms of the relevance contributions, it is possible to single out those residues that differ substantially from the rest by their relevance values. These values, which would commonly be considered to be anomalies or outliers from a data analysis perspective, are instead the most critical residues for model prediction in our study. Positive relevance values contribute to the prediction of the class, whereas negative values counteract the prediction of the class. To highlight those values, we computed the interquartile range (IQR), understood as the difference between the first (Q1) and the third (Q3) quartiles, and used it to establish the lower and upper bounds that will be used to single out the residues with most significant computed relevance values. Table 2 lists such residues, and Figure 5 highlights these most relevant residues at the receptor structure for each conformational state.

More specifically, Table 2a details the most relevant residues for the positive and negative prediction of the active state. It lists the 21 residues of H1 that contributed most in the positive and negative regions of the relevance map of Figure 3a, but also lists another 18 relevant residues from six different regions according to their relevance values. Table 2b focuses on the residues relevant for the prediction of the intermediate state. Such as in the former case of the active state, more than 50% of the residues found as relevant pertain to the H1 region. In particular, there are 21 residues from the H1 region and 18 from four other regions. In the case of the inactive state, Table 2c lists 22 out of 40 residues corresponding to to the H1 region, whereas the remaining 18 belong to five other regions.

**Figure 6:**
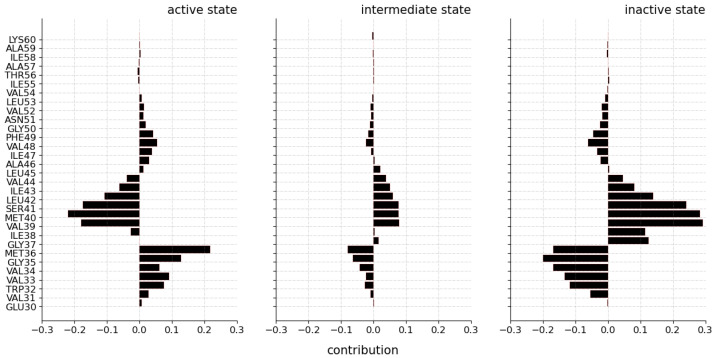
Computed relevance from residues in transmembrane H1 for each conformational state.

A comparison of the residues from all three tables (Table 2) reveals a set of common relevant residues for the different conformational states. Interestingly, a detailed comparison of their relevance values identifies significant differences in their contributions towards the different conformational states. Especially for the case of H1, it was possible to identify subregions of residues (that is, motifs) that alter the positive and negative contribution depending on the predicted state. Regions VAL31^1.31^-MET36^1.35^ have a positive contribution for the active state and negative contributions for the intermediate and inactive state. Region ILE38^1.37^-VAL44^1.43^ shows a prominent negative contribution for the active state, whereas VAL39^1.38^-LEU45^1.44^ and GLY37^1.36^-VAL44^1.43^ contributed to the positive prediction of the intermediate and inactive state, respectively. The same result was found for the residues LEU45^1.44^-VAL52^1.51^, which contribute positively to the prediction of the active state, whereas ILE47^1.46^-VAL52^1.51^ and ALA46^1.45^-LEU53^1.52^ contribute negatively to the prediction of the intermediate and inactive state. From these results, it is possible to state that the 3D positions of the regions VAL31^1.30^-MET36^1.35^ and LEU45^1.44^-VAL52^1.51^ are characteristic of the active state, whereas the 3D positions of region ILE38^1.37^-VAL44^1.43^ are patterns that are distinctive for the intermediate and inactive state and differentiate from the active state. Figure 6 illustrates the aforementioned differences in the H1 residues for the three conformational states.

Focusing now on the remaining regions with lower absolute contributions, it is possible to distinguish the residues LYS140-GLN142 of ICL2 as distinctive for the active state by their positive contributions and as uncharacteristic of the inactive state by their negative contributions. According to their magnitude of contribution, residues MET82^2.53^-GLY83^2.54^ of H2 are characteristic of the inactive state, but uncharacteristic of the active one, whereas PRO88^2.59^ is characteristic of the active state and uncharacteristic of the inactive one. In the case of the intermediate state, residue MET96^2.67^ of H2 was found to be uncharacteristic by a high negative contribution towards the intermediate state; nonetheless, this residue was not highlighted as characteristic for any of the other conformational states. For H4, ASN148^4.40^-LYS149^4.41^ are distinctive for the inactive and intermediate state, whereas they are uncharacteristic of the active state.

From the list of residues meaningfully influencing the three conformational states (Table 2), we can assert that hardly noticeable but distinctive re-orderings happen in the structure, denoting active and inactive states, where most of them are around H1. This result overall confirms the transmembrane helices movement analysis described in the supplementary material of the original work of the dataset under study [52], in which, H1 is reported to be the region with the most substantial movement in the inactive structure. Moreover, there are clear differences between the key residues distinguishing the active state from the intermediate and inactive state, which explains the capability of the classifier to accurately distinguish the active state. For the intermediate and inactive states, similarities in the key residues were found, which, again, helps to explain the difficulties of the classifier in distinguishing these states and the confusion between them (Figure 1). It is important to think of the intermediate states as part of the transformation leading from the inactive towards the active state, for which, according to [52], there are different transition pathways that embrace a multitude of intermediate hardly recognizable conformational states.

#### 2.1.3. Relevance per Receptor Domain

G-protein-coupled receptors have a complex structure in the form of a seven-helix transmembrane (TM) domain, plus the extracellular (EL) and intracellular (IL) domains comprising extracellular and intracellular termini (N-terminus and C-terminus) and loops for connecting the trans-membrane helices [53]. In order to obtain insights at a higher level of abstraction, this study also compared the net contribution of relevance values per region for each conformational state. Table 3 details the total and average relevance contribution of the constituting residues of each region calculated from the relevance values in the 100 randomly selected trajectories. These results explain the relevance per region for the prediction of each conformational state. In particular, Table 3b shows the magnitude of the contribution of the helices in the transition pathway (intermediate state). There, it is cardinal to stress that H6 contributes positively to identifying an intermediate state. Arguably, it is not the region with the highest absolute contribution, but the most meaningful as the activation pathway proceeds via the motion of this helix [52].

**Figure 7:**
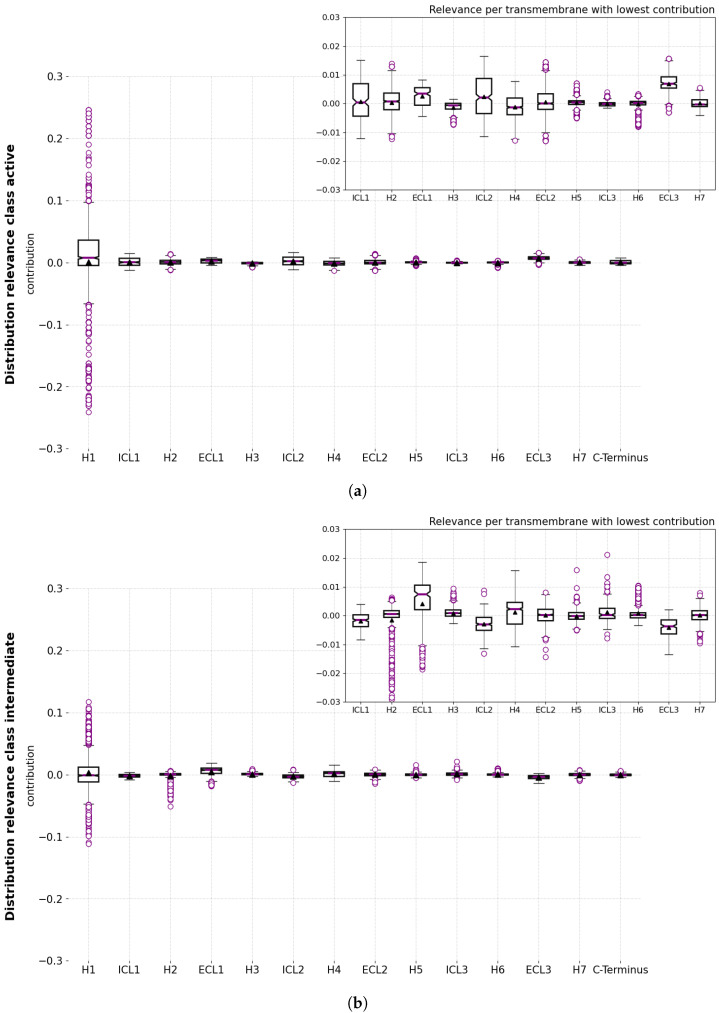
Distribution of the relevance values per residues in the transmembrane helices (H1–H7) and the intracellular (ICL1–ICL3) and extracellular (ECL1–ECL3) loops in the 100 randomly selected trajectories. (**a**) Distribution for the active state. (**b**) Distribution for the intermediate state.

**Figure 8:**
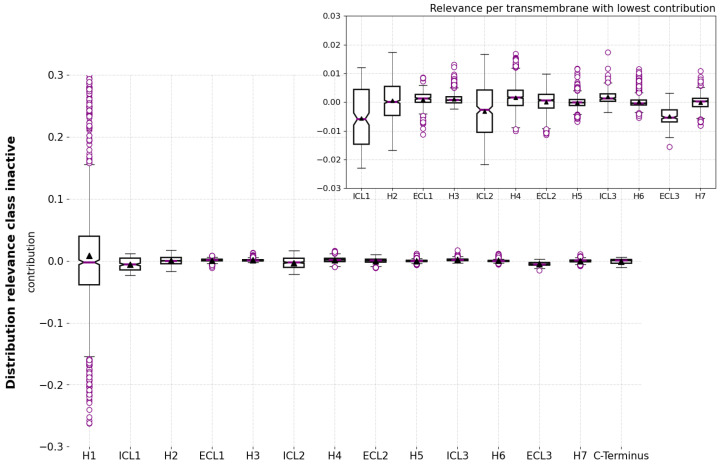
Distribution of the relevance values per residues in the transmembrane helices (H1–H7) and the intracellular (ICL1–ICL3) and extracellular (ECL1–ECL3) loops for the intermediate state.

With the goal of complementing the results in Table 3, the distribution of the relevance values is shown in Figure 7 and Figure 8 for each of the transmembrane and intracellular and extracellular regions. These results show, again, that H1 is the region with the highest contribution magnitude compared with the remaining regions, which are shown at the upper right part of the respective figures. Nonetheless, the box plot representation does not describe a uniform distribution of the relevance values for most regions: in almost all cases, the distributions include outliers that differ from the interquartile range (IQR) by being the most distant points from the mean (black triangle in Figure 7). These are again considered to be the most relevant points for differentiating conformational states, and correspond to the residues described in Table 3.

The existence of both positive and negative outliers in almost all box plots comes from both positive and negative contributing residues in the region. In the case of H1, these phenomena were already explained as due to the fact that this transmembrane region comprises alternating positive and negative contributions (Figure 6). As a consequence, the results of the box plot visualization help us to asses which regions have a non-uniform contribution and may require a further analysis to discern their internal composition regarding the contribution of their residues.

## 3. Materials and Methods

The current study analyzed the MD simulations of the $\beta_{2}AR$ receptor generated on the Google Exacycle platform, as an illustrative example of the proposed methodology. The data included 10,000 parallel simulations, deposited in SimTK, of the inactive (PDB 2RH1) and active (PDB 3P0G) states of the receptor with full agonist BI-167107, carazolol inverse agonist, and free ligand structure (apo). Further details concerning the dataset can be found in [52] and the information about the 3D structure of the inactive state is provided in [54]. Aiming to analyze the transition states to different activation pathways, our study focused on the simulations starting from the inactive state and bound to the full agonist (referred to in this study as *b2ar2rh1-b*). The reason behind this setting relies on the richness of the conformational states space of this structure [55]. We expect that the proposed methodology could easily be replicated for studying other molecular structures.

**Figure 9:**
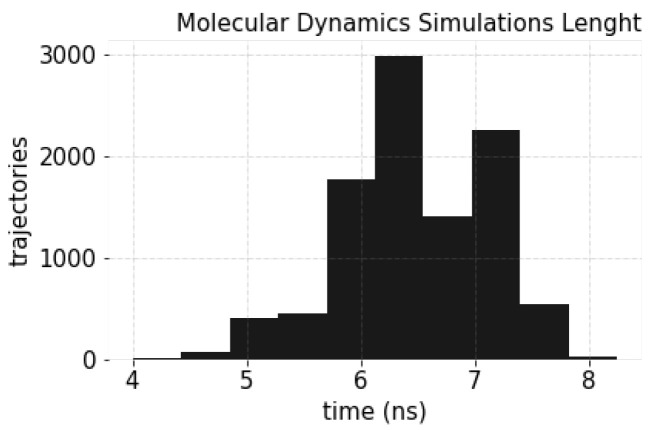
Histogram of MD simulations duration for the $\beta_{2}AR$ receptor with full agonist *BI-167107*.

The distribution of the durations of the simulations is shown in Figure 9. Note that the data comprise multiple short simulations (in the order of a 6 ns average duration), which will be valuable for identifying those *motifs* discerning conformational states that constitute a particular activation pathway. In this context, each simulation step (related to a conformational state) will become an input sample to feed our CNN-based model.

The simulation topology comprised 4640 atoms, 4646 bonds, and 282 amino acids, also referred to as *residues*. Our experiments relied on the MD simulation analysis at the level of residues. In the following subsection, we describe in detail the data transformation into a format that is appropriate for performing predictions using the proposed CNN model.

### 3.1. Data Pre-Processing

This study concerned a classification task of the transition states on the 10,000 raw time series of the MD simulations of the inactive structure with a full agonist.

The center of mass was calculated for each conformational state of the receptor over the trajectory of the MD simulation. This means that, for the 282 residues of the structure, the 3D position of each residue was calculated as the center-of-mass of its constituting atoms. Therefore, our trajectories comprised states of the structure with dimension M×N, where *M* is the number of residues and *N* is the center-of-mass positions (XYZ) of each residue in the 3D space. However, to reduce the complexity of the problem, the dataset was re-dimensioned to a representation of 846 (282 residues × 3 coordinates) × 1 dimensions, instead of using individual information of each coordinate to discern the conformational state.

Regarding the denotation of the receptor states, we followed [52], where four main criteria based on crucial regions of the protein are suggested. In particular, and to distinguish the states, we considered the first criterion: the computation of the distance between Helix 3 (H3) and Helix 6 (H6), measured as the distance between the *alpha-carbon* atoms of the residues arginine 131 and leucine 272 (R131${}^{3.50}$-L272${}^{6.34}$). Therefore, for each frame simulation: if the computed distance is higher than or equal to 14Å, the state of the structure is *active*; if it is lower than or equal to 8.5Å, it refers to an *inactive* state; otherwise, the state is *intermediate*.

**Figure 10:**
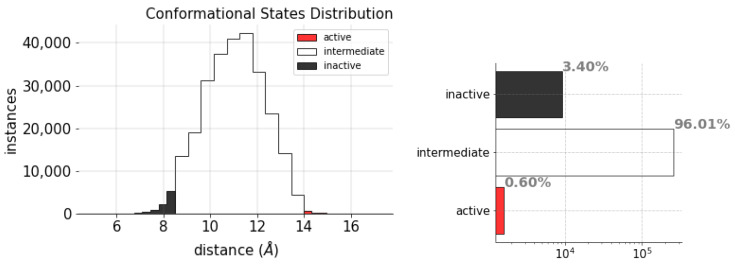
Conformational states distributions per class from the MD trajectories.

Importantly, we must note that the dataset is highly unbalanced, as illustrated by Figure 10. It is evident that the amount of intermediate states in the MD trajectories is much higher than either the inactive or active states. Intermediate states represent 96.01% of the data and the active and inactive states represent, in turn, only 0.60% and 3.40%. This condition could handicap the model’s performance as it might exhibit a bias toward the majority class, ignoring the minority classes. Thus, to avoid this potential limitation, the dataset was randomly under-sampled to the minority class (the active state of the protein). Likewise, to prevent the scale of the variables from affecting the model training, the data were linearly transformed using *min–max* normalization.

### 3.2. Experimental Setup

The MD trajectories were split into two subsets—70% for training the model and the remaining 30% for validation—to ensure that the number of samples was large enough to provide a reliable estimation of the model generalization. In addition, we stratified the splits, i.e., the number of samples per class remained balanced. Table 4 shows the distribution of the data after the undersampling process.

As previously stated, we addressed here a supervised classification problem using CNN models in order to retrieve insights about the most relevant features (understood as *motifs*) in the structure for distinguishing conformational states. CNNs are a particular type of artificial neural network loosely based on the workings of the visual cortex, and represent learning through simple non-linear modules called convolution and pooling for feature extraction and, commonly, a feed-forward network to compute probabilities over the learned features.

In our experiments, we followed an empirical strategy for model architecture definition. This assumes that, given the dimensionality of the data and the limited amount of data available, over-complex architectures would increase the risk of data overfitting. A trade-off was achieved by increasingly adding layers and filters so that the model could learn increasingly more complex attributes. Thus, we clustered the primary layers (*convolution, activation function, and pooling*) into a block for training a shallow architecture (few blocks) with a fixed size of filters, and gradually increased its size until generalization stopped yielding a significant improvement. Table 5 shows different trained architectures used for experimentation. For all cases, the stem cell of our convolution layers considered a kernel size fixed to 3, no padding, and a stride value set to 1. Along with the convolution layers, we established one-dimensional max-pooling layers with a window size of 2 and rectified linear units (ReLU) as activation functions.

The proposed method for defining the 1D-CNN model does not enable applying a large amount of blocks due to the dimension of the data. Adding more blocks would lead to losing relevant information in the feature vector utilized for classifying the conformational states. In addition, it is worth mentioning that increasing the number of blocks beyond four, or removing max pooling layers, have not yielded significant improvements in the accuracy of the model. Therefore, in our experiments, we used an architecture with four blocks and added a *dropout* regularization layer following the first linear layer with a 0.5 value, i.e., 50% of layer neurons randomly become zeros to reduce the model complexity and force it to learn meaningful patterns for the classification. The architecture exhibiting the best results is summarized in Table 6.

In addition to the network architecture design, the training procedure itself also influences the performance of the model. Our training scheme involved experimentation over a wide range of hyper-parameters following the best practices described in [56,57,58,59]. The set of hyper-parameters in our training process involved iterations over 500 epochs using a *mini-batch* strategy with a batch size value of 1024. Cross-entropy was used as a loss function, with adaptive moment estimation (ADAM) as the optimizer, with a 1 × 10^−4^ learning rate and weight decay as regularization set to 1 × 10^−5^. The weight parameters were initialized using the Kaiming uniform distribution proposed by He et al., an established initialization method when working with ReLU activations (see [60]). Algorithms and computations were implemented by using Pytorch 1.10.1 on Python 3.9.7. Likewise, all experiments were conducted on GPUs units from Google Colaboratory platform.

**Figure A1:**
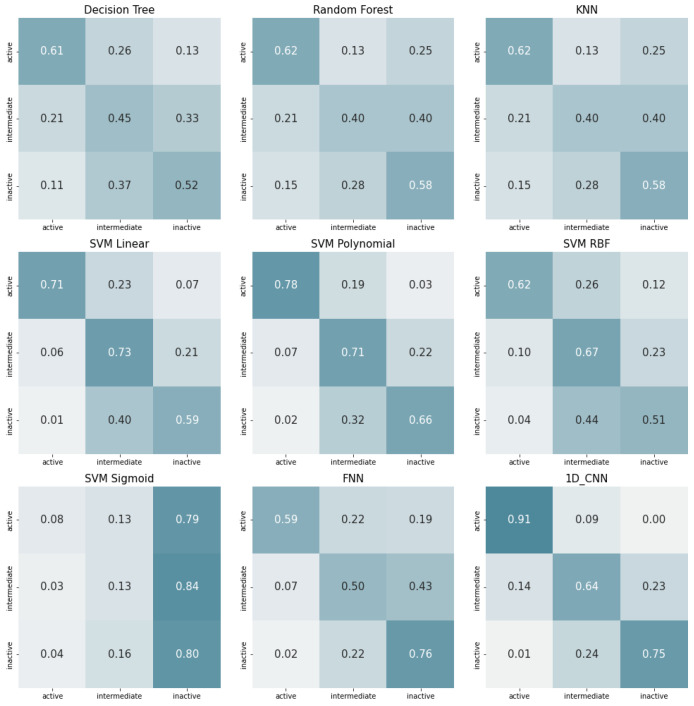
Confusion matrix per ML model.

The classification results of the 1D-CNN-based architecture were also compared against traditional ML approaches. The classification results using a decision tree, random forest, k-nearest neighbors, and support vector machine are described in the Appendix A (Table A1 and Figure A1). Overall, the 1D-CNN-based architecture is shown to clearly outperform the rest of the classifiers in key metrics, even if some methods, such as the SVM with the polynomial kernel, compare reasonably well for given metrics and classes.

## 4. Conclusions

A further understanding of the dynamic properties of protein receptors is critical to the drug discovery process. For this, MD simulations have become primary tools for assessing the underlying mechanisms of biomolecular systems. Nevertheless, an intelligent analysis of the vast amount of generated data remains a critical research challenge aiming to provide more valuable knowledge and promote process efficiency. Machine-learning-based models and, more specifically, DL-based methods, have established themselves, over the last decade, as relevant tools for knowledge generation in this domain. Nonetheless, the DL *black box* limitation—that is, their inherent lack of interpretability—must be addressed, otherwise risking hampering the widespread application of this family of models in relevant domains such as pharmacoproteomics.

In this context, we illustrated our proposed approach to MD data analysis by studying a supervised classification problem of the conformational states (active, intermediate, and inactive) of the MD trajectories from the Google Exacycle Dataset using the inactive structure with full agonist BI-167107. As part of it, a methodology for interpreting the predictions of a 1D-CNN model through the generation of a map of relevant residues for the GPCRs activation pathways using the LRP algorithm was proposed. Notably, the proposed interpretability method produced novel insights by stressing characteristic motifs for the different conformational states from the ML model that would otherwise be hardly recognizable in the transition pathway. In particular, the results of this study provide evidence that the proposed model learned the relationship of crucial residues for differentiating the active and inactive receptor states, whereas such a difference was found to be less clear in the discrimination of the intermediate state. The characteristic motifs might be involved in subtle differences in the movement of transmembrane helices as they were not found to be related to the known motifs relevant in the activation process of the $\beta_{2}AR$ receptor [55]. We expect the proposed approach to be a useful tool for experts for the analysis of MD trajectories by highlighting state-specific characteristic motifs, which might contribute to the pathways of the different conformational states and the transmission of signals to the cell.

Lastly, a further contribution of our study that is worth mentioning is the application of interpretability techniques to a multi-class classification problem. Although interpretability techniques are gaining interest in many interdisciplinary applications, they are often used for binary classification models, where the identification of state-specific features is straightforward based on either positive or negative contributions for the prediction of a given class [37]. In contrast, the LRP interpretability technique was applied here to a multi-class classification model for the prediction of three conformational states. In such a multi-class context, the identification of state-specific residues is not straightforward, as a residue contributes to the prediction of each of the states. To address this difficulty and also to formalize the discovery of contiguous residue regions (*motifs*) with similar contributions, we are currently working on computational methods to single out state-unique features in the context of multi-class classification problems and the use of local neighborhood-aware clustering algorithms to identify contiguous residue regions.

## Figures and Tables

**Table 1:** Summary of classification results. *Support* (last column) refers to the actual number of samples in the class. Macro average (*macro avg*) refers to the harmonic mean of each score reported per class. The reported *accuracy* is described as the proportion of correct predictions out of the total computed predictions.

Class	Precision	Recall	F1-Score	Support
active	0.872154	0.905455	0.888492	550
intermediate	0.671587	0.661818	0.666667	550
inactive	0.780261	0.761818	0.770929	550
macro avg			0.775363	1650
accuracy			0.776364	1650

**Table 2:** Contribution of the residues to each conformational state. First column: residue name; second column: transmembrane region; third column: computed average contribution over 100 randomly chosen trajectories. (**a**) Residues with highly positive and negative computed relevance values for the prediction of the active state when the receptor binds to the full agonist. Listed in order of location in the protein, from H1 to H7. (**b**) Residues with highly positive and negative computed relevance values for the prediction of the intermediate state when the receptor binds to the full agonist. Order as in previous list. (**c**) Residues with highly positive and negative computed relevance values for the prediction of the inactive state when the receptor binds to the full agonist. Order as in previous list.

(a)		
Residue	Region	Average Relevance
VAL31${}^{1.30}$	H1	0.027426
TRP32${}^{1.31}$	H1	0.075927
VAL33${}^{1.32}$	H1	0.092033
VAL34${}^{1.33}$	H1	0.061120
GLY35${}^{1.34}$	H1	0.128148
MET36${}^{1.35}$	H1	0.217308
ILE38${}^{1.37}$	H1	−0.027097
VAL39${}^{1.38}$	H1	−0.178782
MET40${}^{1.39}$	H1	−0.218893
SER41${}^{1.40}$	H1	−0.173772
LEU42${}^{1.41}$	H1	−0.107513
ILE43${}^{1.42}$	H1	−0.062333
VAL44${}^{1.43}$	H1	−0.039133
LEU45${}^{1.44}$	H1	0.011427
ALA46${}^{1.45}$	H1	0.029983
ILE47${}^{1.46}$	H1	0.039122
VAL48${}^{1.47}$	H1	0.054468
PHE49${}^{1.48}$	H1	0.042627
GLY50${}^{1.49}$	H1	0.018640
ASN51${}^{1.50}$	H1	0.011580
VAL52${}^{1.51}$	H1	0.013730
GLU62	ICL1	−0.008478
THR66	ICL1	0.010564
VAL67${}^{2.39}$	H2	0.010418
MET82${}^{2.53}$	H2	−0.008539
GLY83${}^{2.54}$	H2	−0.009841
PRO88${}^{2.59}$	H2	0.008363
SER137	ICL2	−0.008333
LYS140	ICL2	0.010457
TYR141	ICL2	0.008832
GLN142	ICL2	0.014305
ASN148${}^{4.40}$	H4	−0.008919
LYS149${}^{4.41}$	H4	−0.010563
HIS178	ECL2	0.012204
GLN179	ECL2	0.011398
THR195	ECL2	−0.009774
GLN299	ECL3	0.008336
ASP300	ECL3	0.009945
ASN301	ECL3	0.012081
(**b**)		
**Residue**	**Region**	**Average Relevance**
VAL31${}^{1.30}$	H1	−0.009869
TRP32${}^{1.31}$	H1	−0.026707
VAL33${}^{1.32}$	H1	−0.022955
VAL34${}^{1.33}$	H1	−0.043398
GLY35${}^{1.34}$	H1	−0.064005
MET36${}^{1.35}$	H1	−0.079652
GLY37${}^{1.36}$	H1	0.014720
VAL39${}^{1.38}$	H1	0.077509
MET40${}^{1.39}$	H1	0.077171
SER41${}^{1.40}$	H1	0.077424
LEU42${}^{1.41}$	H1	0.059308
ILE43${}^{1.42}$	H1	0.050969
VAL44${}^{1.43}$	H1	0.038719
LEU45${}^{1.44}$	H1	0.020889
ILE47${}^{1.46}$	H1	−0.008240
VAL48${}^{1.47}$	H1	−0.023461
PHE49${}^{1.48}$	H1	−0.016464
GLY50${}^{1.49}$	H1	−0.010800
ASN51${}^{1.50}$	H1	−0.007744
VAL52${}^{1.51}$	H1	−0.008992
ILE94${}^{2.65}$	H2	−0.009512
LEU95${}^{2.66}$	H2	−0.019179
MET96${}^{2.67}$	H2	−0.027799
LYS97	ECL1	−0.013127
TRP99	ECL1	0.008221
THR100	ECL1	0.009572
PHE101	ECL1	0.010311
GLY102	ECL1	0.008605
LYS149${}^{4.41}$	H4	0.010475
ARG151${}^{4.43}$	H4	0.008119
MET156${}^{4.48}$	H4	−0.007606
HIS296${}^{6.58}$	H6	0.008166
ASN301	ECL3	−0.007393
(**c**)		
**Residue**	**Region**	**Average Relevance**
VAL31${}^{1.30}$	H1	−0.054061
TRP32${}^{1.31}$	H1	−0.117340
VAL33${}^{1.32}$	H1	−0.132799
VAL34${}^{1.33}$	H1	−0.167589
GLY35${}^{1.34}$	H1	−0.199008
MET36${}^{1.35}$	H1	−0.168281
GLY37${}^{1.36}$	H1	0.125630
ILE38${}^{1.37}$	H1	0.113863
VAL39${}^{1.38}$	H1	0.291335
MET40${}^{1.39}$	H1	0.283140
SER41${}^{1.40}$	H1	0.241145
LEU42${}^{1.41}$	H1	0.139966
ILE43${}^{1.42}$	H1	0.081891
VAL44${}^{1.43}$	H1	0.046977
ALA46${}^{1.45}$	H1	−0.021858
ILE47${}^{1.46}$	H1	−0.033237
VAL48${}^{1.47}$	H1	−0.060328
PHE49${}^{1.48}$	H1	−0.044268
GLY50${}^{1.49}$	H1	−0.023422
ASN51${}^{1.50}$	H1	−0.016268
VAL52${}^{1.51}$	H1	−0.018806
LEU53${}^{1.52}$	H1	−0.008467
LEU64	ICL1	−0.011626
GLN65	ICL1	−0.018431
THR66	ICL1	−0.013578
VAL67${}^{2.39}$	H2	−0.009970
ASN69${}^{2.40}$	H2	0.010021
TYR70${}^{2.41}$	H2	0.012877
VAL81${}^{2.52}$	H2	0.009884
MET82${}^{2.53}$	H2	0.012388
GLY83${}^{2.54}$	H2	0.013402
VAL87${}^{2.58}$	H2	−0.008223
PRO88${}^{2.59}$	H2	−0.011405
SER137	ICL2	0.010806
LYS140	ICL2	−0.012830
TYR141	ICL2	−0.011080
GLN142	ICL2	−0.016823
ASN148${}^{4.40}$	H4	0.010610
LYS149${}^{4.41}$	H4	0.011207
ASN301	ECL3	−0.008073

**Table 3:** Contribution of the transmembranes (H1–H7) and intracellular (ICL1–ICL3) and extracellular (ECL1–ECL3) loops for each conformational state. Bold highlights correspond to the largest positive and negative contributions. First column: transmembrane region; second column: computed total contribution over 100 randomly chosen trajectories; third column: average contribution. (**a**) Influence of the regions on the active state when the receptor binds to the full agonist. (**b**) Influence of the regions on the intermediate state when the receptor binds to the full agonist. (**c**) Influence of the regions on the inactive state when the receptor binds to the full agonist.

(a)		
Region	Total Relevance	Average Relevance
C-Terminus	0.225551	0.000470
ECL1	0.494248	0.002574
ECL2	0.508710	0.000636
**ECL3**	**1.311195**	**0.006829**
H1	0.810095	0.000817
H2	0.423235	0.000441
**H3**	**−1.352042**	**−0.001243**
H4	−0.947947	−0.001185
H5	0.412987	0.000391
H6	−0.144551	−0.000141
H7	0.202155	0.000263
ICL1	0.132802	0.000692
ICL2	0.764794	0.002390
ICL3	0.013931	0.000087
(**b**)		
**Region**	**Total Relevance**	**Average Relevance**
C-Terminus	−0.047531	−0.000093
ECL1	0.802948	0.004226
ECL2	0.176164	0.000225
ECL3	−0.755840	−0.004042
**H1**	**2.874063**	**0.002823**
**H2**	**−1.374141**	**−0.001508**
H3	1.007901	0.000971
H4	1.063711	0.001259
H5	−0.078420	−0.000073
H6	0.879393	0.000879
H7	0.102290	0.000137
ICL1	−0.389075	−0.001835
ICL2	−0.992810	−0.002903
ICL3	0.205807	0.001204
(**c**)		
**Region**	**Total Relevance**	**Average Relevance**
C-Terminus	−0.294598	−0.000619
ECL1	0.183145	0.000944
ECL2	0.075889	0.000096
ECL3	−0.985589	−0.004903
**H1**	**8.706472**	**0.008884**
H2	0.486780	0.000512
H3	1.181698	0.001047
H4	1.309952	0.001627
H5	−0.121516	−0.000116
H6	0.250733	0.000247
H7	−0.079877	−0.000106
**ICL1**	**−1.107198**	**−0.005592**
ICL2	−1.039449	−0.003269
ICL3	0.316487	0.001907

**Table 4:** Data split distribution per class.

Class	# Training Samples	# Validation Samples
active	1060	550
intermediate	1060	550
inactive	1060	550
Total:	3180	1650

**Table 5:** DL architectures proposed for experimentation. The first column illustrates the number of blocks (convolution, activation function, and pooling) and the total number of filters per block used for experimentation. The second column represents the number of fully connected layers (FCLs) and the amount of neurons used. The third column shows the performance of the architecture in terms of the accuracy.

# Blocks-Filters	# FCL-Neurons	Accuracy
1-64	2-1024-3	0.6339
2-64-128	2-1024-3	0.6830
3-64-128-256	2-1024-3	0.7109
4-64-128-256-512	2-1024-3	0.7375

**Table 6:** CNN architecture proposed.

Layer (Type)	Output Shape	# Parameters
Conv1d-1	[−1, 64, 844]	256
ReLu-2	[−1, 64, 844]	0
MaxPool1d-3	[−1, 64, 422]	0
Conv1d-4	[−1, 128, 420]	24,704
ReLu-5	[−1, 128, 420]	0
MaxPool1d-6	[−1, 128, 210]	0
Conv1d-7	[−1, 256, 208]	98,560
ReLu-8	[−1, 256, 208]	0
MaxPool1d-9	[−1, 256, 104]	0
Conv1d-10	[−1, 512, 102]	393,728
ReLu-11	[−1, 512, 102]	0
MaxPool1d-12	[−1, 512, 51]	0
Flatten-13	[−1, 26,112]	0
Linear-14	[−1, 1,024]	26,739,712
ReLU-15	[−1, 1,024]	0
Dropout-16	[−1, 1,024]	0
Linear-17	[−1, 3]	3075
Total parameters: 13,503,043		
Trainable parameters: 13,503,043		
Non-trainable parameters: 0		

## Data Availability

The dataset under study correspond to molecular structures of the inactive and active states of $\beta_{2}$-adrenergic G protein-coupled receptor simulated on Google Exacycle. The data are publicly available from SimTK (https://simtk.org/projects/natchemgpcrdata). accessed on 2 June 2022.

## Appendix A

Comparative performance results of the 1D-CNN proposed model as a classifier against other traditional ML algorithms are reported next, summarized in Table A1. The related confusion matrices of the different ML model are, in turn, shown in Figure A1.

**Table A1:** Summary of classification results per ML algorithm. Best results are highlighted in bold, providing evidence for 1D-CNN as the best model for predicting conformational states.

Model	Class	Precision	Recall	F1-Score	Accuracy
Decision Tree	active	0.652510	0.614545	0.632959	0.527879
	intermediate	0.418919	0.450909	0.434326	
	inactive	0.527778	0.518182	0.522936	
Random Forest	active	0.636023	0.616364	0.626039	0.530303
	intermediate	0.494331	0.396364	0.439960	
	inactive	0.470414	0.578182	0.518760	
Knn	active	0.542268	0.478182	0.508213	0.430909
	intermediate	0.367213	0.407273	0.386207	
	inactive	0.403604	0.407273	0.405430	
SVM Linear	active	**0.900232**	0.705455	0.791030	0.675152
	intermediate	0.540323	**0.730909**	0.621329	
	inactive	0.682105	0.589091	0.632195	
SVM Polynomial	active	0.893528	0.778182	0.831876	0.716364
	intermediate	0.584708	0.709091	0.640920	
	inactive	0.722222	0.661818	0.690702	
SVM Radial Basis	active	0.811905	0.620000	0.703093	0.601818
	intermediate	0.488095	0.670909	0.565084	
	inactive	0.597046	0.514545	0.552734	
**1D-CNN**	active	0.872154	**0.905455**	**0.888492**	**0.776364**
	intermediate	**0.671587**	0.661818	**0.666667**	
	inactive	**0.780261**	**0.761818**	**0.770929**	
